# Patient with suspected severe acute respiratory syndrome coronavirus 2 infection with successful emergency surgery for ulcerative colitis-associated toxic megacolon

**DOI:** 10.1186/s40792-023-01608-9

**Published:** 2023-02-27

**Authors:** Hirokatsu Hayashi, Jesse Yu Tajima, Ryoma Yokoi, Yuta Sato, Shigeru Kiyama, Takao Takahashi, Naoki Okumura, Yoshihiro Tanaka, Takashi Ibuka, Keisuke Kumada, Masahito Shimizu, Nobuhisa Matsuhashi

**Affiliations:** 1 Department of Gastroenterological Surgery and Pediatric Surgery, Gifu University Graduate School of Medicine, 1-1 Yanagido, Gifu City, 501-1194 Japan; 2 Department of Gastroenterology, Gifu University Hospital, 1-1 Yanagido, Gifu City, 501-1194 Japan; 3 Division of Patient Safety, Gifu University Hospital, 1-1 Yanagido, Gifu City, 501-1194 Japan

**Keywords:** COVID-19, Acute abdomen, Infection control, Inflammatory bowel disease, Pneumonia

## Abstract

**Background:**

In patients with acute severe ulcerative colitis with concomitant severe acute respiratory syndrome coronavirus 2 (SARS-CoV-2) infection, the treatment strategy should consider the presence of pneumonia, respiratory status, and the severity of the ulcerative colitis (UC). We report a case of a 59-year-old man with SARS-CoV-2 infection who was diagnosed with toxic megacolon caused by UC.

**Case presentation:**

Preoperative computed tomography scanning of the chest showed ground-glass opacities. The patient was treated conservatively until the pneumonia improved, but developed bleeding and liver dysfunction associated with UC. As the patient’s condition worsened, emergency surgery with subtotal colorectal resection, ileostomy, and rectal mucous fistula creation was performed while undertaking adequate infection control measures. Intraoperatively, contaminated ascites was observed, and the intestinal tract was markedly dilated and fragile. Nevertheless, the postoperative outcome was positive, with no pulmonary complications. The patient was discharged on postoperative day 77.

**Conclusions:**

The COVID-19 pandemic presented challenges in surgical scheduling. Patients with SARS-CoV-2 infection required close monitoring for postoperative pulmonary complications.

## Background

The coronavirus disease 2019 (COVID-19) pandemic presented a new challenge in the surgical management of patients. The highly contagious nature of the infection necessitated meticulous scheduling or postponement of elective surgeries, such as benign disease or early-stage cancer. Conversely, in cases of advanced cancer or those requiring urgent treatment, surgery was essential while ensuring appropriate infection prevention measures. We describe a case of toxic megacolon with severe acute respiratory syndrome coronavirus type 2 (SARS-CoV-2) infection that required emergency surgery.

## Case presentation

**Fig. 1:**
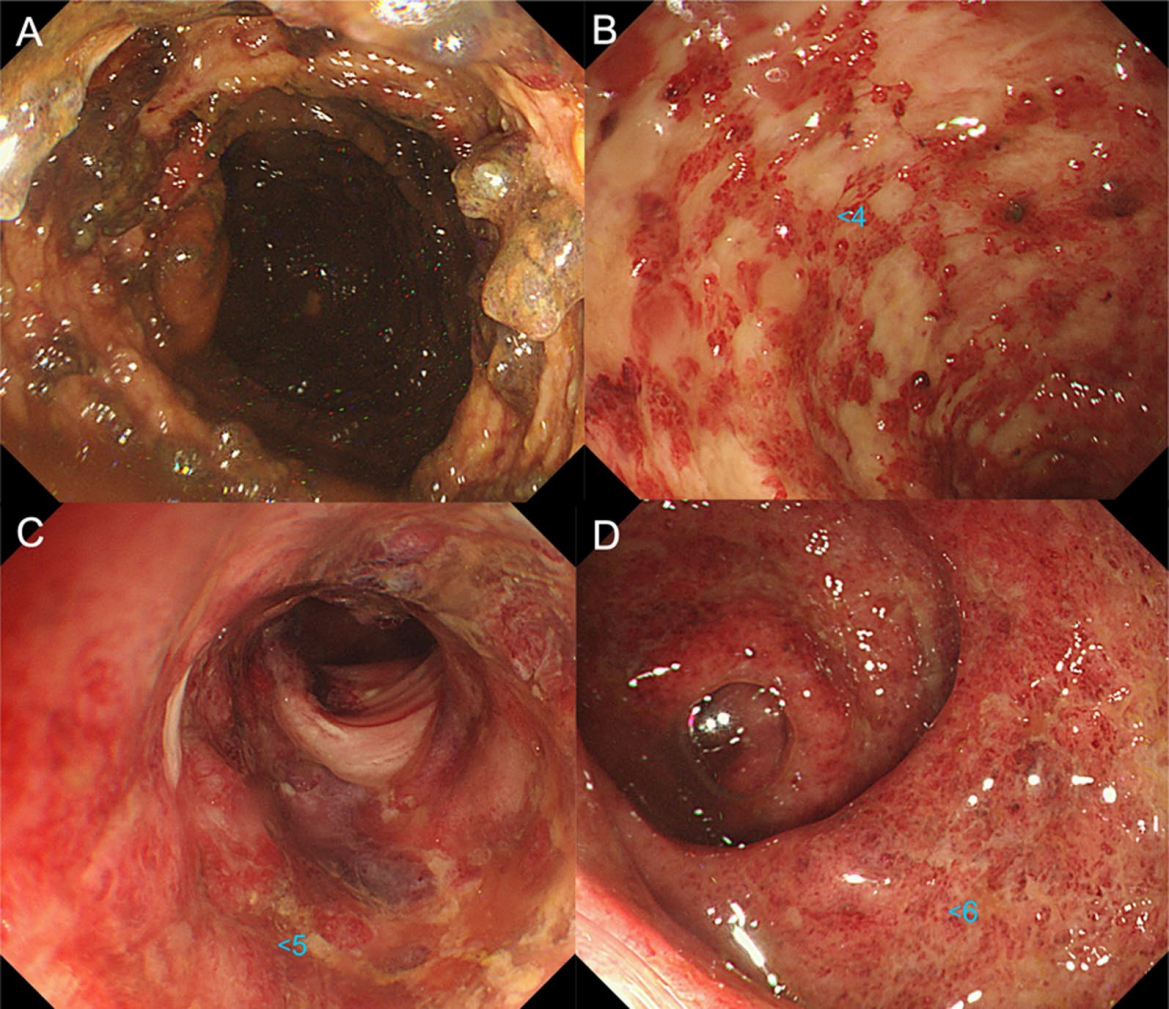
Colonoscopy showing extensive ulceration of the ascending to descending colon and deep-seated ulceration of the sigmoid colon: **A** ascending colon, **B** descending colon, **C** sigmoid colon, **D** rectum

In January 2021, a 59-year-old man presented to a different hospital complaining of abdominal pain and bloody stools. A colonoscopy was performed, and an ulcerative lesion was found in the sigmoid colon. He was diagnosed with ulcerative colitis (UC) and hospitalized. High-dose intravenous steroid therapy with prednisolone (80 mg/day) was initiated. However, 2 weeks after the initiation of prednisolone, exacerbation of the ulcerative lesions from the ascending to the sigmoid colon was found on colonoscopy. Consequently, tacrolimus (4 mg/day) was added, and the prednisone was tapered off week by week (Fig. 1). Two weeks after the initiation of tacrolimus, a further exacerbation of the ulcerative lesion was observed on colonoscopy. On abdominal computed tomography (CT) scans, marked dilatation of the transverse colon was noted; thus, a diagnosis of toxic megacolon was made. Around this time, a nosocomial cluster of SARS-CoV-2 cases occurred at that hospital. The patient was found to have been in close contact with an infected person. However, the patient’s SARS-CoV-2 polymerase chain reaction (PCR) test was negative. Subsequently, he was transferred to our hospital for emergency surgery.

**Fig. 2:**
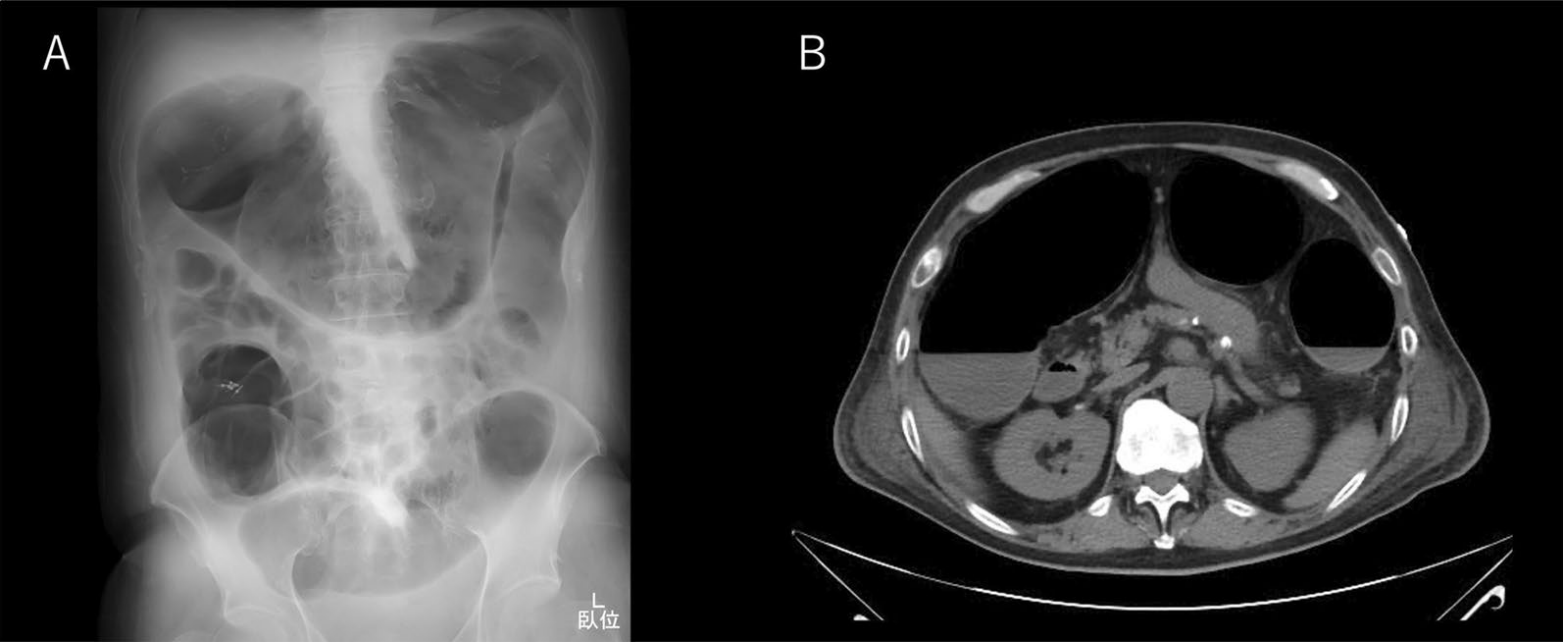
Abdominal radiography (**A**) and contrast-enhanced computed tomography (CT) scan (**B**) obtained at admission: an abdominal radiograph showing a dilated ahaustral transverse colon. Abdominal CT shows diffuse dilatation of the transverse colon up to 80 mm in diameter

**Fig. 3:**
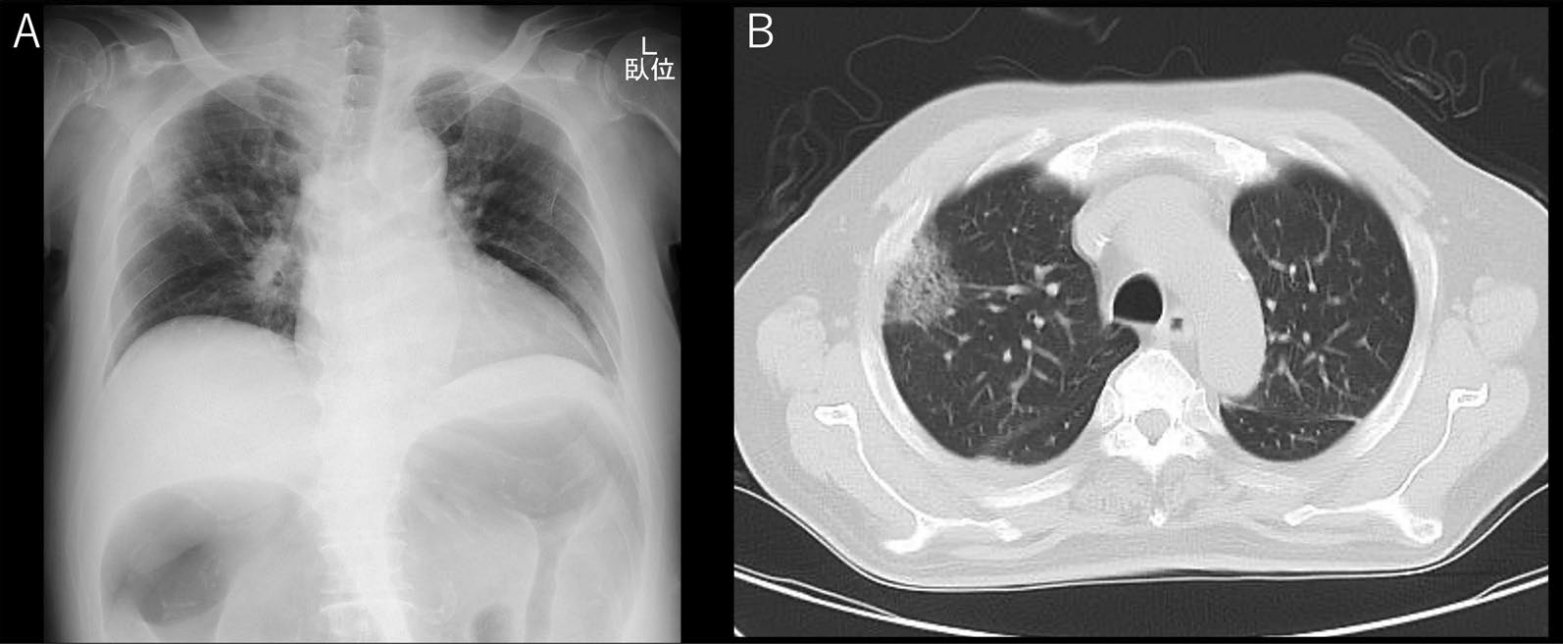
Chest radiography (**A**) and computed tomography (CT) (**B**) images obtained at admission: a chest radiograph showing opacities in the right upper lung field. Chest CT shows peripheral focal ground-glass opacity in the right upper lobes

**Fig. 4:**
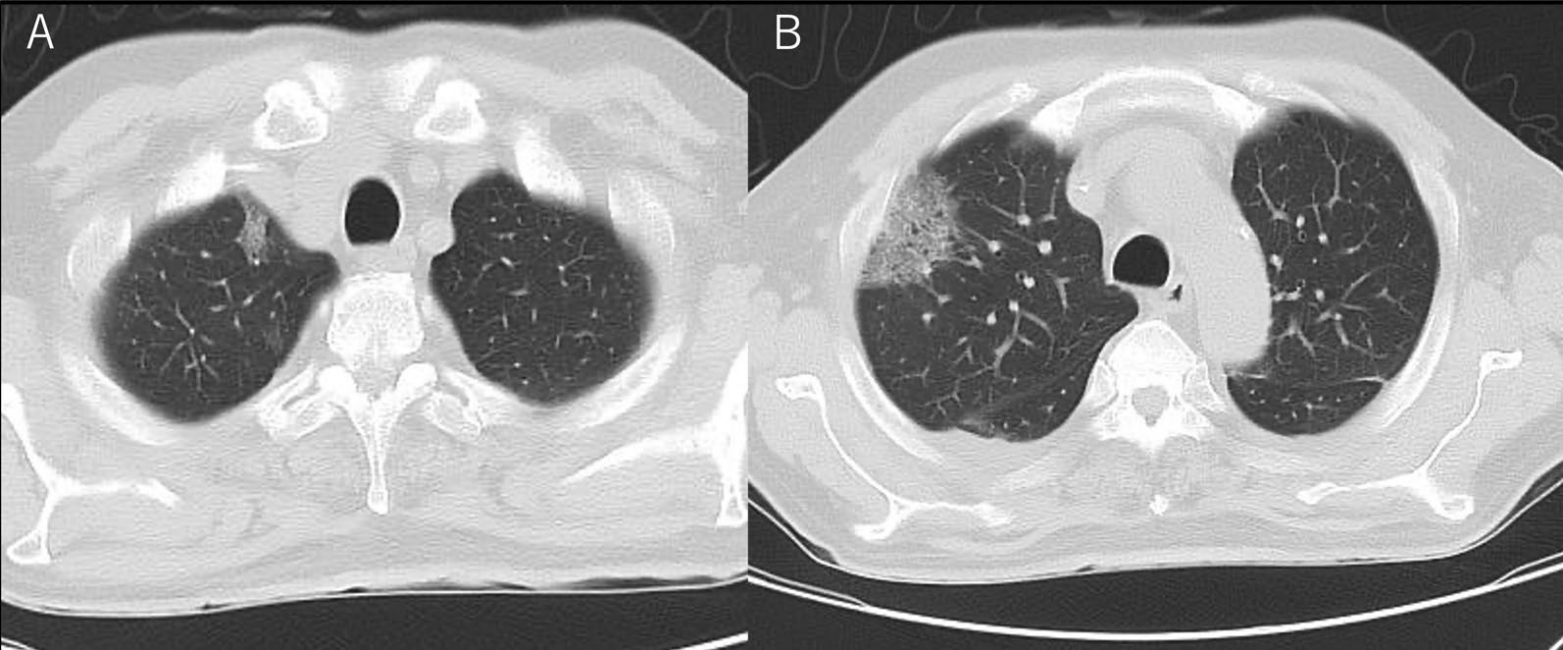
Chest computed tomography scan obtained on the second day after admission. **A** The appearance of a new GGO in the right upper lobe. **B** The existing GGO appears enlarged and shows thickening of the interlobular and intralobular septal walls. *GGO* ground-glass opacity

**Fig. 5:**
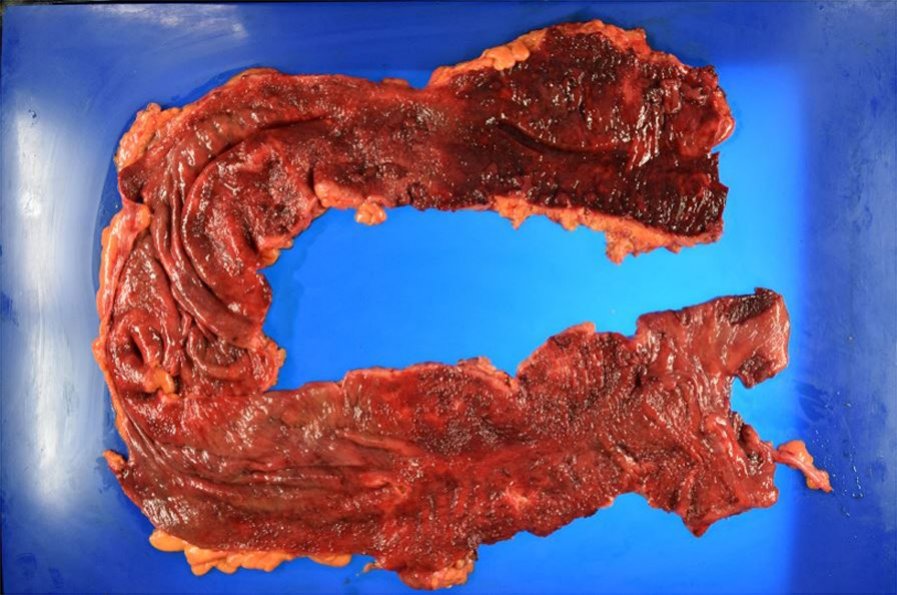
Pathological findings: ulceration and disruption of the glandular ducts, and infiltration of inflammatory cells, mainly lymphocytes, in the intrinsic mucosal layer to the submucosal layer, with particularly severe inflammation of the superficial layer

On arrival, he was conscious and alert, with a blood pressure of 132/93 mmHg, a heart rate of 104 beats/min, a temperature of 36.2 °C, a respiratory rate of 15 breaths/min, and peripheral oxygen saturation of 98% in ambient air. No cardiovascular or respiratory abnormalities were observed. The abdomen was distended but soft, with no signs of peritoneal irritation. The SARS-CoV-2 antigen test was negative. Laboratory tests reported a white blood cell count of 15,000/μL, C-reactive protein level of 12.6 mg/dL, hemoglobin of level 7.9 mg/dL, platelet count of 31.6 × 10^4^/μL, total bilirubin level of 1.8 mg/dL, aspartate transaminase level of 13 IU/L, alanine transaminase level of 19 IU/L, urea level of 19 mg/dL, serum creatinine level of 0.74 mg/dL, and lactate level of 19 mmol/L. Abdominal radiography and contrast-enhanced CT imaging revealed dilatation of the transverse colon, with a bowel diameter of 80 mm (Fig. 2). A chest CT scan showed peripheral ground-glass opacities (GGO) in the right upper lung field (Fig. 3). Based on the patient’s history of SARS-CoV-2 exposure and clinical characteristics typical of pneumonia, it was determined that the patient was infected with SARS-CoV-2. Accordingly, the treatment plan was to schedule the surgery after stabilizing the respiratory symptoms. Treatment for UC continued with prednisone (5 mg/day) and tacrolimus (4 mg/day). Chest CT scans the following day showed new GGO in the right upper lobe, enlargement of the existing GGO, and thickening of the interlobular and intralobular septal walls (Fig. 4). However, the patient’s SARS-CoV-2 PCR test was still negative. On the third hospital day, the patient was found to have persistent bleeding, liver dysfunction, and jaundice, which rendered conservative treatment ineffective. A multidisciplinary conference was held, and emergency surgery was scheduled. The patient underwent a subtotal colectomy and ileostomy, and a rectal mucous fistula was created. Intraoperatively, contaminated ascites was observed, and the intestinal tract was markedly dilated and fragile. Histopathological examination revealed ulceration and disruption of the glandular ducts, and infiltration by inflammatory cells, mainly lymphocytes, into the intrinsic mucosal layer extending up to the submucosal layer, with particularly severe inflammation of the superficial layer (Fig. 5). Postoperatively, the patient was placed on mechanical ventilation in the intensive care unit. On postoperative day (POD) 1, he was extubated owing to improved respiratory parameters. Post-surgery, tacrolimus was discontinued. On POD 2, he was transferred to the infectious ward PCR testing was not performed postoperatively, and the patient was followed up with CT imaging to confirm that there was no exacerbation of pulmonary findings, and isolation was lifted after 14 days. Prednisone (5 mg/day) was administered until POD 3 and then discontinued. On POD 77, the patient was discharged. Eight months post-surgery, the patient underwent a rectal resection, ileorectal anastomosis (ileal J-pouch), and ileal covering stoma, and at 13 months postoperatively, the patient underwent ileal covering stoma closure.

## Discussion

A global epidemic of SARS-CoV-2 infection began in 2019. During the pandemic, management strategies, including surgical treatment, were severely affected. In early 2021, the third wave of SARS-CoV-2 infections overwhelmed healthcare services, and vaccination against SARS-CoV-2 began in Japan. Here, we reported a case of acute severe ulcerative colitis with SARS-CoV-2 infection that was successfully managed by emergency surgery.

This case exhibited a “crazy-paving” pattern characteristic of COVID-19 on chest CT scan. CT scan findings of COVID-19 pneumonia generally show bilateral peripheral multifocal frosted shadows in the early stage of the disease, which change into a “crazy-paving” pattern or infiltrative shadows, 1–3 weeks after the onset of symptoms [1]. The early appearance of GGO is due to multifocal alveolar damage caused by an inflammatory cytokine storm. It is assumed that GGO is caused by the absence of alveolar exudate and edema in the early stage of the disease. Over time the alveolar space fills with exudate and becomes infiltrated [2, 3]. In the present case, unilateral and peripheral GGO with thickening of the interlobular and intralobular septa was observed, known as a “crazy-paving” pattern. These changes were reported to have appeared around one week after the onset of the disease, which is consistent with the time course of COVID-19 disease progression from the time of close contact.

Patients with ground-glass opacities on chest CT scans should be observed even if the RT-PCR test is negative. Yamamoto et al. retrospectively analyzed PCRs performed on 1803 symptomatic or asymptomatic close contacts [4]. They reported that in patients with negative initial PCR tests but with typical findings on CT scans or persistent symptoms, five of 45 cases turned positive after repeated PCR testing.

In this case, it was very difficult to time the surgery correctly. In general, toxic megacolon that develops with ulcerative colitis is an indication for emergency surgery. However, emergency surgery in perioperative SARS CoV-2-infected patients is an extremely high-risk procedure with a reported 51.2% risk of postoperative pulmonary complications and a 23.8% 30-day mortality rate [5]. In this case, a chest CT scan showed deterioration of the primary lesions and the appearance of new lesions, suggesting the possibility of further worsening of symptoms. It was determined that the abdominal symptoms were not urgent and could be treated conservatively; thus, surgery was planned after the pneumonia improved. However, persistent bleeding and liver dysfunction developed during conservative treatment, necessitating emergency surgery.

In the case of elective surgery, if possible, surgery should be delayed for at least 7 weeks after SARS-CoV-2 infection. Further delay may be useful for patients who are still symptomatic more than 7 weeks after diagnosis [6]. Because SARS-CoV-2 RNA is also detected in the feces and urine of patients infected with SARS-CoV-2, caution should be exercised during all procedures involving body fluids. In addition, although the infectivity of the virus is unknown, SARS-CoV-2 RNA can be detected in feces for 3–5 weeks, so caution should be exercised during procedures that open the gastrointestinal tract [7]. When emergency surgery is required, careful postoperative management is necessary because of the high rate of pulmonary complications and mortality due to the possibility of weakened immunity caused by the surgical invasion. In addition, sufficient informed consent should be given before treatment.

It is challenging to identify asymptomatic or mildly symptomatic SARS-CoV-2 carriers by interview or examination alone. If a patient with a subclinical infection is operated on under general anesthesia, SARS CoV-2 may cause serious postoperative complications and further nosocomial infection. Therefore, we perform preoperative reverse transcriptase PCR testing on all patients undergoing elective surgery to prevent infection. In the 2 years since April 2020, 7,239 PCR tests were performed, and preoperative PCR testing was found to be positive in only one pediatric case with asymptomatic infection. The risk of preoperative infection is extremely low in our hospital due to strict behavioral restrictions instituted 2 weeks before surgery.

## Conclusions

We have reported a case of a patient requiring emergency surgery for toxic megacolon and SARS CoV-2 infection control and postoperative management. This case highlights the need to carefully balance the patient’s need for surgery and the risks of surgery while infected with SARS-CoV-2.

## Data Availability

The data can be accessed from PubMed (https://pubmed.ncbi.nlm.nih.gov) or the Japan Medical Abstracts Society (https://search.jamas.or.jp).

## Abbreviations

SARS-CoV-2: Severe acute respiratory syndrome coronavirus 2
UC: Ulcerative colitis
COVID-19: Coronavirus disease 2019
CT: Computed tomography
PCR: Polymerase chain reaction
GGO: Ground-glass opacities
POD: Postoperative day
